# Is Surveillance Endoscopy Necessary after Colectomy in Ulcerative Colitis?

**DOI:** 10.5402/2011/509251

**Published:** 2011-07-02

**Authors:** Yasutaka Shuno, Keisuke Hata, Eiji Sunami, Masaru Shinozaki, Kazushige Kawai, Tetsu Kojima, Giichiro Tsurita, Masaya Hiyoshi, Nelson H. Tsuno, Joji Kitayama, Hirokazu Nagawa

**Affiliations:** ^1^Department of Surgical Oncology, The University of Tokyo, Tokyo 113-8655, Japan; ^2^Department of Surgery, The Institute of Medical Science, The University of Tokyo, Tokyo 108-8639, Japan; ^3^Department of Surgery, Kanto Rosai Hospital, Japan Labour Health and Welfare Organization, Kanagawa 211-8510, Japan; ^4^Department of Transfusion Medicine, The University of Tokyo, Tokyo 113-8655, Japan

## Abstract

The role of surveillance endoscopic followup in colectomized patients with long standing total colitis is controversial. Here, we aimed to clarify its usefulness for the early detection of dysplasia and cancer in this group of patients. Ninety-seven colectomised UC patients followedup by surveillance endoscopy were retrospectively investigated by reviewing the pathological reports. Patients had received either subtotal colectomy and ileo-rectal anastomosis (IRA) or total proctocolectomy and ileal anal anastomosis (IPAA). Definite dysplasia was diagnosed in 4 patients, who had received IRA; among them, 2 were carcinoma with submucosal invasion, and one was a high-grade dysplasia. Postoperative surveillance endoscopy is useful for the detection of early cancer in the remaining colonic mucosa of UC patients, and those receiving IRA, in which rectal mucosa is left intact, would be good candidates. However, its effectiveness for patients receiving IPAA, in which the rectal mucosa is resected, needs further investigation.

## 1. Introduction

Long-standing extensive ulcerative colitis (UC) is reported to be a risk factor for the development of colorectal cancer (CRC) [1–3]. Surveillance colonoscopy instead of prophylactic proctocolectomy is generally recommended for those with total colitis for more than 8 years after the onset or left-sided colitis for more than 15 years [4, 5].

Subtotal colectomy with ileo-rectal anastomosis (IRA) had been the surgical treatment of choice for UC until pouch operation was established, but patients undergoing subtotal colectomy have also been reported to carry a certain risk of developing carcinoma in the rectal remnant [6, 7]. Furthermore, Johnson et al. reported that most of them were found in an advanced stage [7]. Although the importance of surveillance colonoscopy for the rectal remnant has been emphasized, few reports describe the effectiveness of surveillance colonoscopy in the colectomized population. 

Since the 1980s, total proctocolectomy and ileal pouch-anal anastomosis (IPAA) has become the surgical treatment of choice for UC [8, 9]. Although total proctocolectomy eliminates the risk of colorectal cancer, several cases with cancer in the rectal remnant or ileal pouch have been reported. The major methods of IPAA are stapled IPAA without mucosectomy and handsewn IPAA with mucosectomy. Stapled IPAA is a safer and less complicated method than handsewn IPAA, but the rectal remnant of a few centimeters may retain a malignant potential. Mucosectomy could theoretically remove all the rectal mucosa that might have malignant potential. However, the resected specimens of the patients undergoing pouch excision following mucosectomy revealed that isolated rectal mucosa might remain [10, 11]. Indeed, several cases of “rectal” carcinoma after mucosectomy have been reported [12–14]. Moreover, several cases of dysplasia or cancer in the ileal pouch have been reported after IPAA [15–17].

Although those cases of UC who have undergone colectomy may be at risk of carcinoma in the rectal remnant or the ileal pouch, the effectiveness of surveillance endoscopy after colectomy is still controversial. The aim of this study is to clarify the effectiveness of surveillance endoscopy after colectomy in UC.

## 2. Methods

### 2.1. Patients

Ninety-seven UC patients who received colectomy and postoperative surveillance endoscopy in our surgical department, in the period between January 1979 and December 2008, were retrospectively analyzed. Among them, 29 had received IRA, and 68 IPAA (stapled without mucosectomy in 47 and hand-sewn with mucosectomy in 21).

### 2.2. Endoscopy

Surveillance endoscopy, using flexible endoscopes, was conducted regularly in most of the cases, and in addition to the conventional observation by the experienced colonoscopist, the dye spray method was performed for the better visualization of mucosal lesions. Only those patients who had at least one postoperative biopsy were included. Biopsy specimens were taken from the lesions suggestive of dysplasia as well as from the apparently normal flat rectal mucosa of patients who had received IRA or stapled IPAA (either with or without mucosectomy). Pathological reports from all patients were retrospectively reviewed for the patients' clinicopathological features, the surgical procedures, and the colonoscopic and histological diagnosis.

### 2.3. Pathology

Dysplasia was graded, according to the Riddell' classification, into high-grade (HGD), low-grade (LGD), indefinite (IND), or negative for dysplasia [18].

### 2.4. Evaluation

Histopathological reports were retrospectively reviewed, and the patient's clinicopathological features, such as age, duration after onset of UC, time after colectomy, and histopathological diagnosis of the resected surgical specimen, were analyzed according to the presence or absence of dysplasia.

## 3. Results

**Figure 1:**
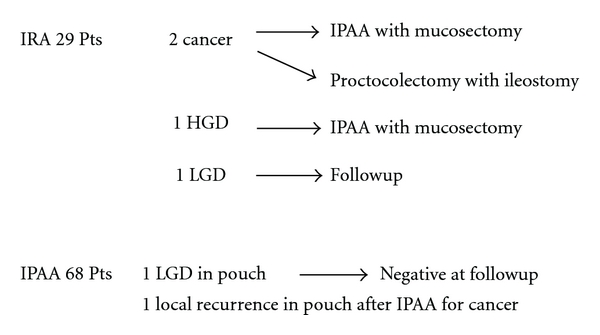
Results of postoperative surveillance endoscopy after colectomy in patients with ulcerative colitis.

A total of 531 surveillance endoscopies were performed. The median followup time after operation was 5.3 and 15.6 years for those who had received IPAA and IRA, respectively. Results of postoperative surveillance endoscopy are summarized in Figure 1. 

**Figure 2:**
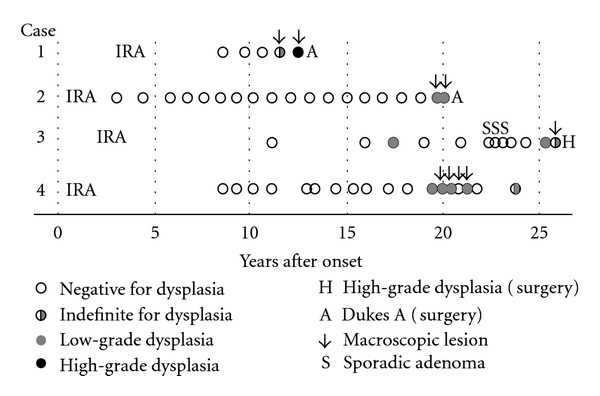
Results of surveillance colonoscopy in patients with definite dysplasia after ileo-rectal anastomosis.

By the surveillance endoscopy, 4 patients who had received IRA were diagnosed as definite dysplasia (Table 1, Figure 2). Among them, 2 had received hand-sewn IPAA with mucosectomy, and one rectal excision. One patient was not operated on due to the presence of various extracolonic complications, but the subsequent surveillance endoscopy revealed no abnormalities. Histopathological examination of the resected specimen revealed adenocarcinoma invading the submucosa in 2 of them, and in 1, HGD was diagnosed. 

One patient, who had received hand-sewn IPAA, had an ulcerative lesion in the staple line of the pouch, and was diagnosed as LGD. However, the subsequent surveillance revealed no abnormalities. No other patients receiving IPAA had definite dysplasia or cancer detected during the postoperative surveillance.

One patient, who had received IPAA with mucosectomy due to colitic cancer, was diagnosed as HGD during the pouch surveillance conducted 2 years after the operation. The ileal pouch was surgically resected, and the permanent ileostomy was left. The histopathology revealed transmurally invasive signet-ring cells, which was compatible with recurrence of the colitic cancer. No other dysplastic or neoplastic lesions were found during the pouch surveillance.

## 4. Discussion

It is well recognized that patients receiving subtotal colectomy remain at a risk of developing carcinoma in the remnant rectum; most of the carcinomas of the remnant rectum are diagnosed in an advanced stage. The development of rectal cancer is reported to be associated with the duration of ulcerative colitis and with poor surveillance, and most patients who developed rectal cancer presented with an advanced tumour stage (III and IV) [19]. In our series, by the performance of a meticulous surveillance colonoscopy, definite dysplasia and cancer were found in four (14%) out of 29 patients who had received IRA. Analysis of the resected specimens revelead carcinoma with submucosal invasion in two of them, and HGD in one. Therefore, surveillance colonoscopy was considered effective for the detection of dysplasia or cancer at an earlier stage.

Stapled IPAA is a safer method, with lower incidence of complications than hand-sewn IPAA, but the rectal remnant of few centimeters will theoretically retain the risk of malignant transformation [15]. In a study conducted by the British group, involving 135 patients who had received IPAA with double-stapling technique (DST), and were followed by surveillance colonoscopy for a median period of 56 months [20], no cases of dysplasia or carcinoma were found; thus, it was concluded that cuff surveillance in the first decade after IPAA with DST is not necessary. On the other hand, a study from the Cleveland Clinic diagnosed 2 LGD and 4 HGD among the 178 patients who had received stapled IPAA [21]. They concluded that patients with dysplasia of the colon or the upper third of the rectum can be efficiently managed with stapled IPAA, provided that a postoperative surveillance program is adequately indicated. In our series, no cases of dysplasia of the remnant rectum were found among the patients who had received stapled IPAA. Therefore, taking together our results and those previously reported, we can speculate that the risk of carcinoma development in the remnant rectum is relatively low. On the other hand, although not common, carcinoma or dysplastic lesion may occur in those patients who had received stapled IPAA, since remnant rectum is left similar to those who receive IRA. Although the risk of carcinoma of the remnant rectum is relatively low, we believe that surveillance colonoscopy should be performed in those patients who had received stapled IPAA. The early detection of carcinoma or dysplastic lesion by the surveillance program will allow the indication of anal sphincter saving procedures, such as the pouch advancement method.

Mucosectomy is a technique to remove all the rectal mucosa with malignant potential. In our series, no cases of dysplasia of the rectal remnant were found among those patients who had received hand-sewn IPAA. However, the histological analysis of the resected specimens of patients who had received pouch excision following mucosectomy revealed the presence of remaining isolated rectal mucosa [10, 11]. Moreover, several cases of “rectal” carcinoma after IPAA with mucosectomy have been reported [12–14]. Therefore, mucosectomy is not enough to completely remove the rectal mucosa, in order to eliminate the risk of cancer.

Recently, the risk of carcinoma of the ileal pouch is a major concern among colorectal surgeons. In our series, one patient who had received stapled IPAA was diagnosed as LGD of the staple line ulcer of the pouch, but the subsequent surveillance endoscopic followup of this case revealed no remaining dysplastic lesions. Gullberg et al. found 5 cases of dysplasia in the ileal pouch by the surveillance endoscopy, and all these cases had persistent severely atrophic mucosa [13]. Several reports have shown the detection of dysplasia or cancer in the ileal pouch or in the rectal remnant after restorative proctocolectomy [21, 22]. However, several authors consider that the risk of carcinoma development in the ileal pouch is not high. Hulten et al. conducted a 30-year followup study of the Kock pouch, and concluded that it was very unlikely for invasive carcinoma to develop in the ileal pouch [23]. Thompson-Fawcett et al. examined a retrospective cohort consisting of 1221 patients with ileal pouches, and found only one patient with LGD, who had never had pouchitis [24]. Herline et al. found only one LGD out of 222 biopsies from the ileal pouch [25]. Although the risk of carcinoma development in the ileal pouch is controversial, it is of note that most of the patients with carcinoma of the ileal pouch were diagnosed at an advanced stage. We consider that the appropriate surveillance endoscopy should be indicated for those patients receiving IPAA, until definitive conclusions can be drawn.

In summary, postoperative surveillance endoscopy for UC is useful to detect cancer at an early stage, which will allow the inidication of curative restorative proctocolectomy. Patients receiving IPAA are those who mostly should receive surveillance endoscopy. Although the total risk of cancer development in patients who had undergone IPAA seems to be relatively low, surveillance should be indicated until further investigations deny its effectiveness.

## Figures and Tables

**Table 1:** Cases of definite dysplasia or cancer after ileo-rectal anastomosis.

Case	Age at onset	Age at IRA	IRA duration	Grade of dysplasia at endoscopy	Postoperative diagnosis	Survival
1	29	33	9	HGD	sm	Alive
2	57	58	19	LGD	sm	Alive
3	22	24	23	LGD	m(HGD)	Alive
4	56	58	20	LGD	—	Alive

HGD: high-grade dysplasia: IRA, ileo-rectal anastomosis: LGD: low-grade dysplasia;M:  mucosa; sm: submucosa.
